# An eHealth symptom and complication management program for cancer patients with newly created ostomies and their caregivers (Alliance): a pilot feasibility randomized trial

**DOI:** 10.1186/s12885-023-10919-x

**Published:** 2023-06-10

**Authors:** Shenmeng Xu, Xianming Tan, Chunxuan Ma, Rebecca S. McElyea, Karl Shieh, Angela M. Stover, Angela Smith, Karyn Stitzenberg, Ethan Basch, Lixin Song

**Affiliations:** 1 School of Nursing, University of North Carolina, Chapel Hill, North Carolina USA; 2 UNC Lineberger Comprehensive Cancer Center, Chapel Hill, NC USA; 3 Gillings School of Global Public Health, UNC-CH, Chapel Hill, NC USA; 4 School of Medicine, UNC-CH, Chapel Hill, NC USA

**Keywords:** Ostomy, Quality of life, Randomized controlled trial, Self-management, eHealth, Cancer, Complication

## Abstract

**Background:**

Cancer patients with newly created ostomies face complications that reduce quality of life (QOL) and increase morbidity and mortality. This proof-of-concept study examined the feasibility, usability, acceptability, and initial efficacy of an eHealth program titled the “Patient Reported Outcomes-Informed Symptom Management System” (PRISMS) during post-ostomy creation care transition.

**Methods:**

We conducted a 2-arm pilot randomized controlled trial among 23 patients who received surgical treatment with curative intent for bladder and colorectal cancer and their caregivers. After assessing QOL, general symptoms, and caregiver burden at baseline, participants were randomly assigned to PRISMS (*n* = 16 dyads) or usual care (UC) (*n* = 7 dyads). After a 60-day intervention period, participants completed a follow-up survey and post-exit interview. We used descriptive statistics and t-tests to analyze the data.

**Results:**

We achieved an 86.21% recruitment rate and a 73.91% retention rate. Among the PRISMS participants who used the system and biometric devices (*n* = 14, 87.50%), 46.43% used the devices for ≥ 50 days during the study period. Participants reported PRISMS as useful and acceptable. Compared to their UC counterparts, PRISMS patient social well-being scores decreased over time and had an increased trend of physical and emotional well-being; PRISMS caregivers experienced a greater decrease in caregiver burden.

**Conclusions:**

PRISMS recruitment and retention rates were comparable to existing family-based intervention studies. PRISMS is a useful and acceptable multilevel intervention with the potential to improve the health outcomes of cancer patients needing ostomy care and their caregivers during post-surgery care transition. A sufficiently powered RCT is needed to test its effects.

**Trial registration:**

ClinicalTrial.gov ID: NCT04492007. Registration date: 30/07/2020.

**Supplementary Information:**

The online version contains supplementary material available at 10.1186/s12885-023-10919-x.

## Introduction

Ostomy creation—a procedure that allows bodily waste to pass through a surgically created opening (i.e., the stoma) for elimination—occurs while treating patients with bladder, colorectal, and gynecological cancer (e.g., ovarian, cervical, and uterine cancer) [1, 2]. Patients often encounter complex, interrelated symptoms and complications (e.g., dehydration, skin problems, and infection) during post-ostomy creation recovery [3, 4], leading to reduced quality of life (QOL) [5–7] and high rates of morbidity and mortality [8, 9]. Poor symptom management is a primary reason for emergency room visits [10–12], readmissions [4, 9, 13], and high costs of care for patients with ostomies [4]. While caregivers often provide help with household chores, transportation, and personal care, having an ostomy surgery is a life-changing event for both patients and their family caregivers [14, 15]. Yet, there are limited interventions that focus on empowering ostomates and caregivers to monitor and manage symptoms and complications, improve QOL, and reduce preventable hospital visits during the transition from in-patient, professional care to post-treatment, in-home self-management.

To better meet ostomates’ needs during this transitional time, our multidisciplinary team developed the Patient Reported Outcomes-Informed Symptom Management System (PRISMS), which is an eHealth symptom and complication monitoring and self-management program for cancer patients with new ostomies and their caregivers. We analyzed data extracted from the social media platform Reddit; qualitative interviews with Wound, Ostomy, and Continence care Nurses (WOCN) and other clinicians for cancer patients with ostomies [16]; and a caregiver survey [17]. We rigorously tested the usability of a PRISMS prototype through focus groups and interviews among stakeholders, including cancer patients with newly created ostomies (*n* = 6), their caregivers (*n* = 3), and clinicians (*n*= 8). Based on stakeholders’ input and the results of our initial usability testing [18], we refined the content, navigation, functionality, and appearance of PRISMS, and the intervention implementation protocol.

Guided by the modified stress-coping model [19, 20], we conducted a proof-of-concept study to achieve the following aims: 1) Determine the feasibility of implementing PRISMS in clinical care among cancer patients with newly created ostomies and their caregivers; 2) Evaluate the usability and acceptability of PRISMS; and 3) Assess the magnitude of benefit (initial efficacy) of PRISMS on patient and caregiver QOL and symptoms, caregiver burden, and patient healthcare services utilization.

## Methods

### Study design

We performed a 2-arm pre- and post-randomized controlled trial (RCT) among cancer patients and their primary caregivers (dyads) (ClinicalTrial.gov ID: NCT04492007). Following Institutional Review Board approval (#19–2205), we recruited eligible patients and caregivers and obtained consent to participate in our study. Patient-caregiver dyads were randomly assigned to either PRISMS or the usual care (UC hereafter) at a 2:1 (PRISMS:UC) ratio using permuted block randomization after each participant completed their baseline (T1) surveys. Randomization was stratified by ostomy type (colostomy, ileostomy, and urostomy) because ostomy type is related to post-surgical recovery and outcomes. After participating in the intervention for 60 days—the period when patients are at high risk for complication-related ER visits and readmissions [21]—participants completed a follow-up (T2) survey and an optional post-exit interview.

### Participants

Eligible patients had to have a primary caregiver and discharged from the hospital after receiving a newly created ostomy for treating colorectal, bladder, ovarian, cervical, or uterine cancer with curative intent within the past month. Caregivers were eligible based on willingness to participate and did not receive a cancer diagnosis or treatment during the study to ensure the patient-caregiver dyad efforts were focused on the patient’s care. Both patients and caregivers were ≥ 18 years old and could read and speak English.

### Procedures

The research team met with potentially eligible patients and caregivers identified from the Urology, Gastrointestinal Surgery, and Surgical and Gynecologic Oncology inpatient and outpatient clinics located in a tertiary care academic medical center to provide study information and screen for interest and eligibility. After separately obtaining written informed consent from both patients and caregivers, participants completed the surveys via phone or with an individualized REDCap link online, based on preference. Post-exit interviews were conducted via phone with participants who agreed to provide feedback on the study. All data were collected by trained research assistants. The PI (Song) and the research assistants were blinded to participant group assignments. For each participant who completed the T1 survey, we provided a $20 gift card study incentive. T2 survey study incentives included a Fitbit tracker and a smart water bottle for the patients, and a Fitbit tracker for the caregivers.

### Usual care (UC)

The UC for patients with newly created ostomies and their family caregivers included the distribution of printed materials and ostomy care demonstrations provided by clinical nurses prior to hospital discharge. We provided the contact information for the research nurse to the participants assigned to the UC group to contact for professional support if necessary.

### Intervention

Participants accessed the Web-based PRISMS using their own electronic devices or a loaned iPad with a data plan from the research team. First, PRISMS included information written in plain language and demonstrative videos that provided education and skills training on ostomy care, the most common complications (e.g., dehydration and skin problems), safe physical activity, and fall prevention for optimized recovery. Second, PRISMS provided patients and caregivers with personalized feedback on care and support based on the symptom and complication severity and dispatched detailed self-care-management instructions based on the continuous monitoring of patient-reported outcomes (PRO) (e.g., fatigue), evaluated by the National Institutes of Health (NIH) published Common Terminology Criteria for Adverse Events (CTCAE) with scores ranging from 0–10, and data from the smart devices (e.g., steps, sleep, weight, and fluid intake) [22]. Patients and caregivers who reported mild symptoms and complications (scores ranging from 0–4) received self/family-management affirmation and encouragement to continue the strategies. For moderate symptoms (e.g., fatigue score ranging from 4–7, temperature > 100.8**°**F), patients and caregivers received self-care instructions and a follow-up phone call from our interventionist WOCN. Patients and caregivers who reported severe symptoms (scores ranging from 8–10) were directed to contact their treatment team at the hospital or to call 911 (note: the WOCN helped to contact the treatment team when needed). Finally, to facilitate social support, the “Talk with a Nurse” function provided participants with the ability to contact the WOCN for professional support. PRISMS also provided access to an online forum to help participants communicate with other patients and caregivers in the study who may face similar challenges for peer support. The online forum was moderated by research staff to prevent harassment and animosity. Caregivers could use PRISMS to access the education modules, view the patient PRO survey feedback, and view the digital device data for both the patient and their own.

### Measurement

#### Feasibility

Recruitment and retention rates were assessed based on the data obtained from administrative tracking records. We aimed to recruit 18 patient-caregiver dyads from 29 or fewer approached dyads (60.00%) and retain 60.00% of the recruited dyads.

#### Usability

We measured usability with a usability survey [23] (Appendix 1) containing 19 items on a 5-point Likert scale. Participants who completed at least 80.00% of the questions were included to evaluate the three aspects of program usability: general, content, and navigation. Also, a participation satisfaction questionnaire with 9 items on a 5-point Likert scale evaluated patient and caregiver satisfaction with various aspects of the study and intervention. In the PRISMS group, we examined user activity logs and digital devices data to evaluate usage patterns. Upon completion of the T2 survey, we conducted post-exit interviews with the participants in both groups to obtain feedback on acceptability, usefulness, and recommendations for improvements.

#### Initial efficacy

Patients and caregivers separately completed a series of psychometrically sound questionnaires at T1 and T2. QOL was measured using the 27-item Functional Assessment of Chronic Illness Therapy General Scale (FACT-G) for patients [24] and for caregivers [25]. We calculated the FACT-G total score and scores of the physical, social/family, emotional, and functional subdomains. PROMIS questionnaires assessed general symptoms, including the severity of pain [26], sleep disturbance [27], and the frequency of anxiety [28], depression [29], and fatigue [30]. We used the Zarit Burden Interview to measure self-reported caregiver burden [31]. Finally, we measured healthcare utilization by extracting the number of ostomy-related ER visits, readmissions, referrals, or follow-up visits during the 90 days after T1 baseline assessment from patient medical charts.

### Data analysis

Study feasibility was evaluated using recruitment (number of patient-caregiver dyads that consented divided by the total number of dyads approached) and retention (the percentage of dyads retained during the study period) rates and based on the hypothesis test for recruitment and retention rates (pre-set goals: both at 60%). We examined the usability and satisfaction survey data, as well as PRISMS and device usage data with means and standard deviations. Thematic summaries were derived from the qualitative data collected from participants during post-exit interviews of the participants conducted in both the PRISMS and UC groups.

To evaluate initial efficacy, we used t-test to examine the group differences in the score changes between the T1 and T2 surveys. All randomized participants were included with an intent-to-treat analysis. We also examined the demographic differences between the PRISMS and UC groups using the Fisher test (categorical variables) and the unpaired Student’s t-test (continuous variables). Considering this is a pilot study and the small sample size, we did not adjust for multiple comparisons. We used Cohen's d to assess the clinically meaningful differences (d = 0.2 small effect; d = 0.5 medium effect; and d > 0.8 large effect). All statistical analyses were performed using SAS (version 9.4, SAS Institute, Inc., Cary, NC) at the 0.05 significance level [32], and *p* < 0.1 was considered as marginal significance.

## Results

### Participant characteristics

After completing the T1 survey, dyads were assigned to PRISMS (*n* = 16) and UC (*n* = 7) groups (Table 1). Male patients accounted for 75.00% of the PRISMS group and 71.43% of the UC group. Of the caregivers in the PRISMS and UC groups, 37.50% and 57.14% were spouses, respectively. Most caregivers were female, 68.75% in the PRISMS group and 100% in the UC group (*p* = 0.27). By ostomy type, 12 patients had urostomies (*n* = 8 PRISMS and *n* = 4 UC), 8 had colostomies (*n* = 5 PRISMS and *n* = 3 UC), and 3 had ileostomies (all PRISMS). All patients (100%) in the UC group and 50% of the patients in the PRISMS group were unemployed (*p* = 0.052).

**Table 1:** Participant demographics

Characteristics	Patients					Caregivers				
	PRISMS (*N* = 16)		UC (*N* = 7)			PRISMS (*N* = 16)		UC (*N* = 7)		
	N	%	N	%	*P*-value	N	%	N	%	*P*-value
Gender										
Male	12	75.00	5	71.43	1.00	5	31.25	0	0.00	0.27
Female	4	25.00	2	28.57		11	68.75	7	100.00	
Race										
White	12	75.00	5	71.43	0.60	13	81.25	5	71.43	0.70
Black	2	12.50	2	28.57		2	12.50	2	28.57	
Other	2	12.50	0	0.00		1	6.25	0	0.00	
Education										
Bachelor's degree or above	7	43.75	2	28.57	0.66	9	56.25	1	14.29	0.09
Others	9	56.25	5	71.43		7	43.75	6	85.71	
Patient-Caregiver Relationship										
Spouse	6	37.50	4	57.14	0.65	-	-	-	-	-
Others	10	62.50	3	42.86		-	-	-	-	-
Marriage status $${}^{a}$$										
Single	8	50.00	2	28.57	0.41	4	25.00	5	71.43	1.00
Long-term partner	8	50.00	5	71.43		12	75.00	2	28.57	
Employee status										
Employed	8	50.00	0	0.00	0.052	9	56.25	3	42.86	0.67
Unemployed	8	50.00	7	100.00		7	43.75	4	57.14	
Income										
Less than $90,000	11	68.75	6	85.71	1.00	12	75.00	6	85.71	1.00
More than $90,000	4	25.00	1	14.29		3	18.75	1	14.29	
Don't know/refused	1	6.25	0	0.00		1	6.25	0	0.00	
Religious										
Ever	15	93.75	7	100.00	1.00	12	75.00	7	100.00	0.67
Never	0	0.00	0	0.00		3	18.75	0	0.00	
Refused	1	6.25	0	0.00		1	6.25	0	0.00	
Cancer type										
Colorectal	8	50.00	3	42.86	1.00	-	-	-	-	-
Bladder	8	50.00	4	57.14		-	-	-	-	
Ostomy type										
Urostomy	8	50.00	4	57.14	0.68	-	-	-	-	-
Ileostomy	3	18.75	0	0.00		-	-	-	-	
Colostomy	5	31.25	3	42.86		-	-	-	-	
	Mean	SD	Mean	SD	*P*-value	Mean	SD	Mean	SD	*P*-value
Age	56.81	13.73	57.71	25.81	0.93	48.06	16.30	38.00	27.47	0.28
Number of people supported by income	2.47	1.68	1.86	0.38	0.20	2.88	1.54	2.00	1.00	0.19

*UC*, Usual Care, *SD* standard deviation

^a^Single included widowed, divorced, and separated; Long-term partner included girlfriend/boyfriend/partner married or domestic partnership

### Feasibility

#### Recruitment and retention rates

**Fig. 1:**
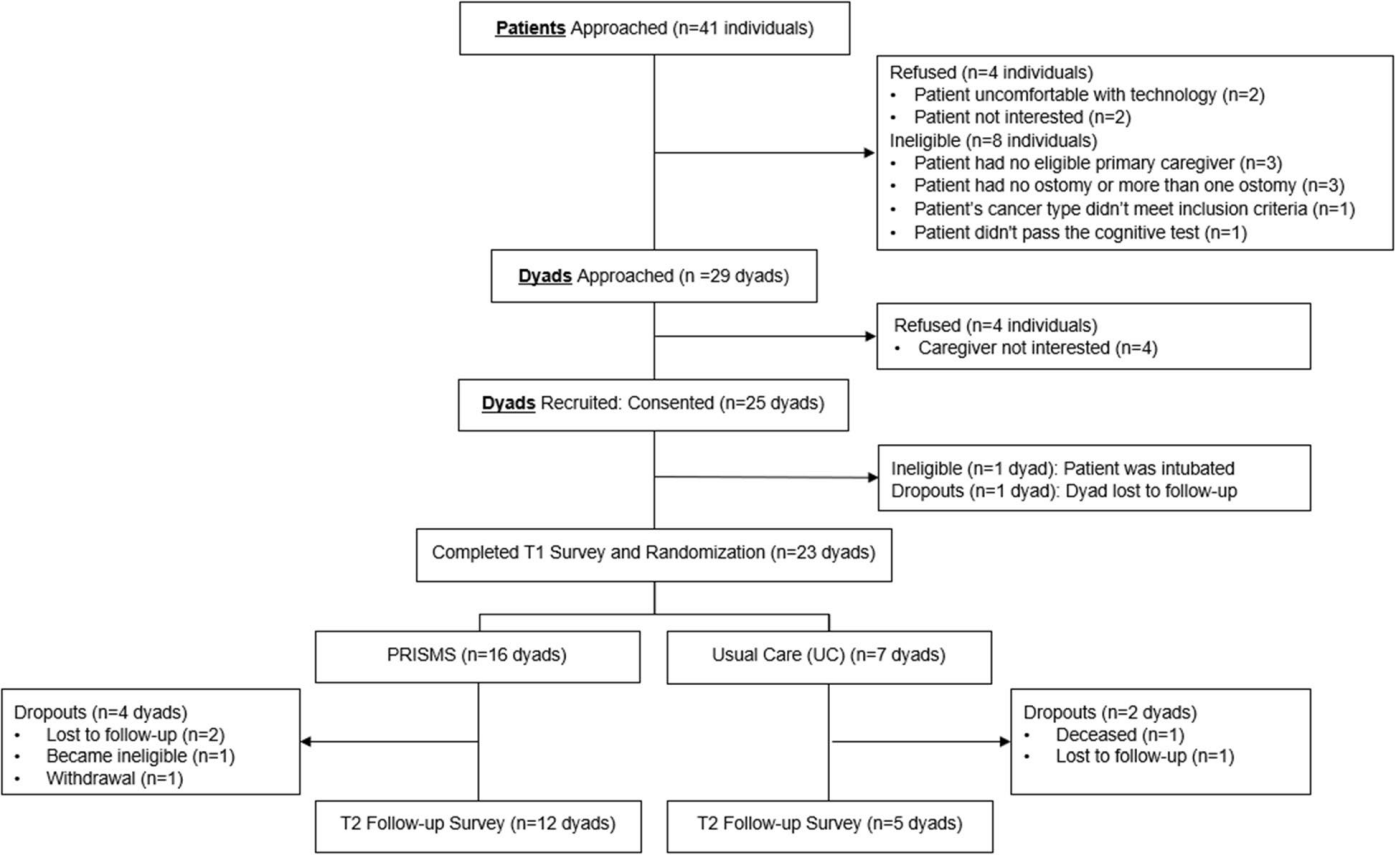
Flowchart of the Study

Between November 2020 and July 2021, 29 of the 41 patients we approached were eligible and interested in the study; 25 dyads consented, achieving a recruitment rate of 86.21% (95% CI: 72.86%-99.56%); 23 dyads (46 individuals) completed the T1 survey and randomly assigned to PRISMS or UC. Seventeen dyads completed the T2 survey, resulting in a retention rate of 73.91% (95% CI: 53.5%-87.4%). The two dyads who consented but did not complete T1 survey were excluded from the retention rate calculation (Fig. 1). Both recruitment and retention rates exceeded our preset goal of 60% for recruitment and 60% for retention, yet the hypothesis tests indicated we could not reject the null hypothesis (*p* = 0.002 and *p* = 0.12 for recruitment and retention rates, respectively) at the pre-specified alpha level 0.05.

The main reasons for refusal to participate in the study were due to the individual discomfort with technology (*n* = 2) or patient/caregiver disinterest (*n* = 6). Other reasons for ineligibility included patients not meeting caregiver, ostomy, or cognitive test criteria. Lost contact was the main reason for dropping out of the study. One patient in the UC group died before T2.

### Usability

#### Usability survey

As shown in Table 2, PRISMS caregivers (*n* = 11) reported significantly greater scores in program/resource navigation compared to the UC caregivers (*n* = 4) (*p* = 0.01), indicating that PRISMS was easier for caregivers to use than the printed materials provided to the UC group. Although not statistically different, PRISMS participants (*n* = 12 patients and *n* = 11 caregivers) reported more positive scores about the general program and the PRISMS content compared to participants in the UC group (*n* = 4 dyads).

**Table 2:** Usability and satisfaction survey results

	PRISMS			UC		
		Mean	SD	Mean	SD	*p*-value
**Usability**						
General	Patient	20.17	3.66	17.00	3.65	0.16
	Caregiver	20.36	3.20	17.50	2.08	0.12
Program content	Patient	20.71	3.41	19.75	4.99	0.67
	Caregiver	21.36	3.83	18.50	3.11	0.20
Program navigation	Patient	38.16	6.37	35.69	7.46	0.53
	Caregiver	37.45	7.24	30.75	1.89	0.01
**Satisfaction**						
Time took to review the program every time	Patient	4.08	0.79	3.20	1.10	0.08
	Caregiver	3.83	0.72	3.20	1.48	0.41
Ease of navigating the program	Patient	4.08	1.00	3.60	1.34	0.42
	Caregiver	4.00	0.95	4.20	0.84	0.69
Information received from the program	Patient	4.00	0.95	3.40	1.14	0.28
	Caregiver	3.92	0.90	4.00	1.00	0.87
Quality of the information	Patient	4.08	1.00	3.60	0.89	0.36
	Caregiver	4.00	0.85	4.20	1.10	0.69
Program increased the knowledge about managing symptoms and complications	Patient	4.08	1.00	3.80	1.10	0.61
	Caregiver	4.17	0.72	3.60	1.14	0.23
Program improved methods of managing symptoms and complications	Patient	3.75	1.22	3.60	0.89	0.81
	Caregiver	4.17	0.72	3.40	1.52	0.33
Program improved methods of communication with my family about cancer-care-related, sensitive topics	Patient	3.17	1.40	3.40	0.89	0.74
	Caregiver	4.00	0.95	3.60	1.14	0.47
Time and effort taken to complete questionnaires	Patient	3.83	1.19	3.20	1.48	0.37
	Caregiver	3.64	1.12	2.40	1.14	0.06
Program improved knowledge about the effects of relationships with my family and caregiver	Patient	3.64	1.29	3.80	0.84	0.80
	Caregiver	3.92	0.79	3.60	1.67	0.70

*UC* Usual Care, *SD* standard deviation

#### Participant satisfaction

PRISMS patients (*n* = 12) reported marginally significant greater mean satisfaction scores with the time spent on reviewing their respective programs than patients in the UC group (*n* = 5) (*p* = 0.08). Moreover, compared with caregivers in UC group, PRISMS caregivers reported marginally significant greater mean satisfaction scores on the questionnaires (*p* = 0.06).

#### PRISMS and device usage

**Fig. 2:**
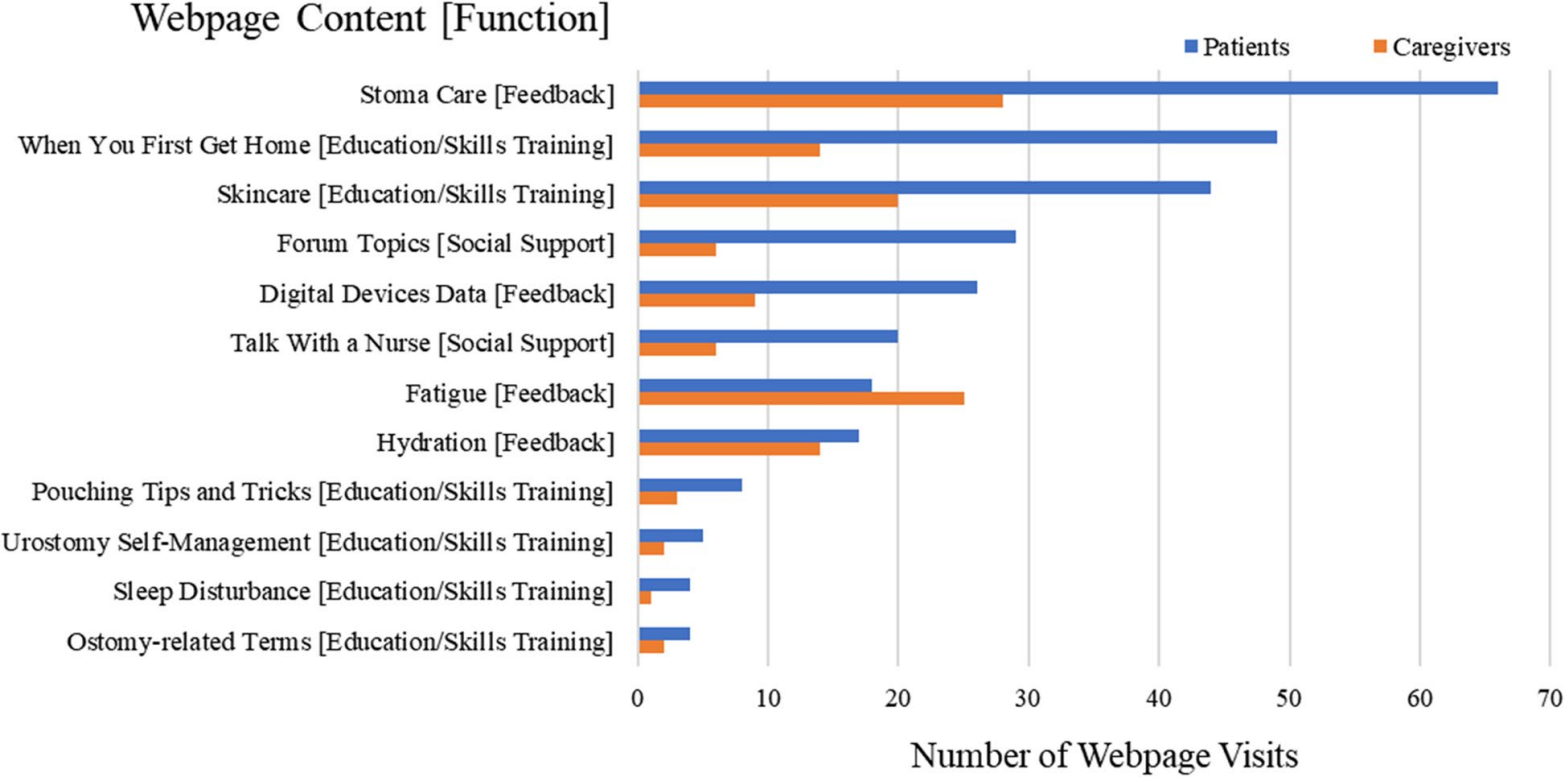
Most frequently visited PRISMS webpages during the intervention period

As displayed in Fig. 2, both patients and caregivers most frequently visited PRISMS pages were “Stoma Care [Feedback]” and “Skin Care [Education/Skills Training].” Additionally, patients visited “When You First Get Home [Education/Skills Training],” and caregivers visited “Fatigue [Feedback].” Among PRISMS participants (*n* = 16 dyads/*n* = 32 individuals), 28 participants (87.50%) used and synced the smart devices to the PRISMS program at least once, and 13 participants (46.43%) used and synced the devices for more than 50 days. During the 60-day intervention period, patients used the smart scale for an average of 34.64 days (SD = 15.55), the smart bottle for an average of 43.64 days (SD = 21.02), and the Fitbit to track steps and sleep for an average of 45.57 days (SD = 16.84) and 43.14 days (SD = 16.97), respectively. Caregivers used Fitbit for step tracking for 34.86 days (SD = 18.90) and sleep tracking for 34.69 days (SD = 19.47).

#### Post-exit interview

PRISMS participants (*n* = 6) found that PRISMS was a useful resource immediately after hospital discharge, but usefulness waned over time. While the UC participants (*n* = 5) considered the self-care pamphlets provided by the hospital useful, the participants also felt the pamphlets were too technical and lacked tailored information relevant to patient cancer type and needs. UC participants also desired more self-management skills demonstrations. Although the research nurse was available to both PRISMS and UC participants as needed, PRISMS participants emphasized that the research nurse was the most well-liked aspect of the program, including her experience with ostomy, rapport, and ability to provide information.

### Initial efficacy

#### Cancer-related QOL and general symptoms

Table 3 displays the results of group differences in the mean score changes between the T1 and T2 surveys and between the PRISMS and UC participants. Overall, we observed inconsistent patterns of change across different sub-scales. Compared to UC patients, PRISMS patients exhibited larger but not significant changes in the FACT-G physical (diff = 0.36) and emotional well-being scores over time (diff = 0.16). We also observed smaller, but nearly significant changes (*p* < 0.10) in the FACT-G total score (diff = -11.02) and FACT-G functional well-being score (diff = -5.21). PRISMS patients reported a larger decrease in the FACT-G social well-being score than UC patients. (diff = -6.10, *p* = 0.02). In caregivers, we observed larger but not significant (*p* > 0.10) changes in all FACT-G scores (differences range: 0.16–5.31), except for functional well-being (diff = -1.29), over time in the PRISMS group than in the UC group. Additionally, PRISMS caregivers experienced a larger decrease (diff = -7.37, *p* = 0.09) in the Zarit Burden Interview than UC caregivers. The clinically meaningful differences in patient FACT-G social and functional well-being scores and in the Zarit Burden Interview score in caregivers were large.

**Table 3:** The Initial efficacy of PRISMS on health outcomes

		Patients									Caregivers								
		T1		T2		T2-T1		Score difference between PRISMS and UC			T1		T2		T2-T1		Score difference between PRISMS and UC		
Outcome	Group	Mean	SD	Mean	SD	Mean	SD	Mean	Cohen's d	*P*-value	Mean	SD	Mean	SD	Mean	SD	Mean	Cohen's d	*P*-value
QOL																			
FACT-G Total Score^†^																			
	PRISMS	74.40	18.65	81.85	16.10	7.45	8.98	-11.02	-1.05	0.07	77.80	13.86	82.15	10.61	4.35	8.16	5.31	0.57	0.30
	UC	68.70	13.13	87.13	11.19	18.43	13.59				66.18	12.79	65.21	19.86	-0.97	11.71			
FACT-G Physical Well-being^†^																			
	PRISMS	16.92	7.08	23.28	5.12	6.36	5.51	0.36	0.07	0.89	21.97	4.25	22.35	2.95	0.39	1.86	3.19	1.10	0.21
	UC	18.00	6.32	24.00	5.20	6.00	3.16				18.98	4.63	16.18	6.5	-2.8	4.69			
FACT-G Social Well-being^†^																			
	PRISMS	23.24	4.96	20.82	6.45	-2.42	3.20	-6.10	-1.41	0.02^*^	20.83	5.46	22.25	4.33	1.42	2.84	0.58	0.16	0.77
	UC	20.97	3.95	24.53	2.36	3.57	6.29				18.60	5.98	19.43	6.13	0.83	5.15			
FACT-G Emotional Well-being^†^																			
	PRISMS	17.25	3.6	18.73	3.82	1.36	1.63	0.16	0.07	0.93	15.75	3.65	17.58	2.15	1.83	3.46	2.83	0.93	0.10
	UC	17.20	3.7	18.4	1.95	1.20	3.70				14.20	3.83	13.2	4.55	-1.00	1.41			
FACT-G Functional Well-being^†^																			
	PRISMS	17.00	7.15	19.45	6.12	2.45	5.16	-5.21	-1.13	0.05	19.25	5.26	19.96	5.2	0.71	4.22	-1.29	-0.31	0.57
	UC	12.53	5.36	20.2	4.55	7.67	2.72				14.40	3.13	16.40	5.32	2.00	3.94			
General Symptoms^‡^																			
Anxiety																			
	PRISMS	51.08	15.74	47.46	8.96	-3.62	13.42	4.86	0.41	0.46	58.96	8.04	50.43	7.09	-8.53	7.79	-2.65	-0.38	0.48
	UC	53.16	11.01	44.68	8.04	-8.48	6.56				59.40	6.34	53.52	8.68	-5.88	3.49			
Depression																			
	PRISMS	48.47	9.75	46.83	6.84	-1.63	8.55	3.97	0.38	0.49	48.08	9.61	48.69	8.96	0.62	6.52	0.84	0.12	0.82
	UC	48.02	13.93	42.42	5.84	-5.6	14.77				53.08	8.44	52.86	10.82	-0.22	7.29			
Pain																			
	PRISMS	65.01	11.64	49.41	9.41	-15.6	13.02	-10.32	-0.88	0.12	44.64	10.16	44.14	7.26	-0.50	4.54	-2.76	-0.47	0.40
	UC	54.60	10.62	49.32	8.51	-5.28	7.28				54.32	9.16	56.58	12.84	2.26	8.65			
Fatigue																			
	PRISMS	55.08	13.45	49.32	7.32	-5.76	10.86	0.42	0.04	0.94	44.73	10.77	46.13	7.59	1.39	9.59	-3.59	-0.31	0.57
	UC	49.12	8.92	42.94	7.65	-6.18	8.73				51.10	15.01	56.08	13.82	4.98	16.07			
Sleep																			
	PRISMS	57.58	11.92	50.34	6.83	-7.24	8.15	0.22	0.03	0.96	51.87	9.17	48.19	8.14	-3.68	8.83	-3.32	-0.43	0.44
	UC	54.44	3.91	46.98	7.59	-7.46	6.54				53.56	4.55	53.20	6.76	-0.36	3.47			
Zarit caregiver burden ‡																			
	PRISMS	-	-	-	-			-		-	15.36	9.88	11.58	11.29	-3.77	6.57	-7.37	-0.95	0.09
	UC	-	-	-	-						17.60	8.91	21.2	16.54	3.60	10.38			

Footnote:

*QOL* quality of life, *FACTG* The Functional Assessment of Cancer Therapy – General, *UC* Usual Care, *SD* standard deviation, *CL* Confidence limits

^a^^*^: *P*-value ≤ 0.05 is considered significant. *P*-value ≤ 0.1 is considered marginal significance

^b^^†^: Higher scores indicated more positive results

^c^^‡^: Higher scores indicated more negative results

#### Healthcare utilization

During the 90 days after the dyad completed the baseline survey (T1), patients in the PRISMS group, on average, visited the hospital 3.69 (SD = 4.98) times for lab tests and treatments (e.g., blood draw, radiation, chemotherapy, nutrition, X-ray, CT scan, MRI, etc.) and 8.88 times (SD = 4.13) for provider consultations. Comparatively, UC patients had more frequent lab tests and treatments (Mean = 7.29, SD = 11.09) and provider visits (Mean = 10.14, SD = 4.98). Yet, the group differences did not reach statistical significance (*p* = 0.29 and *p* = 0.53).

## Discussion

We conducted this proof-of-concept RCT to examine the feasibility, usability, and acceptability of the PRISMS—a multilevel eHealth symptom and complication monitoring and self-management program—for cancer patients with newly created ostomies and their caregivers. We also evaluated the preliminary efficacy of PRISMS on the health outcomes of patients and their caregivers. Findings from this study will guide the refinement of PRISMS and the study protocol for a sufficiently powered efficacy RCT.

It is worth noting that this proof-of-concept pilot study was conducted in 2020–2021 and the COVID-19 pandemic may have affected the process and results. During this time, the number of COVID-19 cases fluctuated, particularly when the vaccine became available. Most UC participants were in the study when the number of positive COVID-19 cases decreased and the vaccinated population increased in North Carolina (between 3/17/21 and 8/1/21), whereas most PRISMS participants were in the study when the number of COVID-19 cases surged (before 3/17/21 or after 8/1/21). Participants in the PRISMS and UC groups may have experienced different levels of COVID-19-related physical, emotional, social, and functional sequelae that affected QOL and symptoms, but impossible to be teased out in this proof-of-concept study given the small sample size.

### Feasibility

Among the 23 dyads (46 individuals) who completed the T1 survey, six dyads failed to complete the assessment at T2, including one deceased, one had stoma reversal, one withdrew from the study due to other life burdens, and three lost to follow-up. Although we used strategies suggested in other intervention studies to retain patients and caregivers [33] (e.g., providing gift cards and gifts after study completion; following up using various approaches), participants had challenges, competing demands, and other priorities in life during the pandemic, leading to their withdrawal from the study. But providing multiple means of survey data collection have been identified by participants as helpful, e.g., online survey is more flexible than telephone survey.

Although we could not reject the null hypothesis at the previously specified (and stringent for a pilot study) alpha level 0.05, the overall *p*-value (0.12) indicates that such a study is likely feasible. Based on the useful data from this proof-of-concept study, it is feasible to conduct an efficacy two-arm RCT with 173 patient-caregiver dyads with a 3-year recruitment. Specifically, we would need to contact approximately 201 dyads in 3 years, or 34 dyads per year per site for a two-site study and retain 128 dyads to the end of study to achieve 80% power to detect a moderate effect size of 0.5. In order to achieve 80% power to detect a small effect size of 0.2, we will need approximately 800 patient-caregiver dyads and retain 592 dyads in a four-site study, which is feasible to recruit in 4 years (50 dyads per site per year). In future testing, we plan to address the main reasons for refusal to participate by improving our research activity flow and enhance retention by refining study protocol to ensure potential participants understand that PRISMS has a low technology literacy requirement and emphasize utility during post-surgical care transition; we will also elaborate and emphasize the importance of supporting research to improve clinical care. However, it is worth noting that our recruitment rate (~ 86%) is comparable with the rates reported in many family-based intervention studies (range = 8%-100%) but higher than the median enrollment rate (23%) across these studies [33]; our retention rate (73.91%) was also favorable compared to the average retention rates (69%, range = 16–100%) reported in a recent systematic review [33].

### Usability

Our comprehensive assessment with usability and satisfaction surveys and qualitative interviews indicated that our eHealth PRISMS intervention was acceptable, accessible, and of interest to cancer patients and caregivers. Usability has been identified as a critical component of good practice in developing eHealth programs to reduce user cognitive workload, complement working memory limitations, and provide visual/immersive learning [34, 35]. Managing a newly created stoma at home requires knowledge and skills which can be provided and enhanced by eHealth programs such as PRISMS. Approximately 88% of PRISMS participants used the system and the digital devices, but only 46% used the devices for ≥ 50 days during the study period, indicating that patients and caregivers would likely use the support for symptom and complication management when they transitioned from professional care to post-surgical in-home self-management. Decrease in use may reflect those patients and caregivers became comfortable with the ostomy and had fewer concerns and questions over time.

### Initial efficacy

The decreases in the FACT-G social and functional well-being scores, which contributed to decrease in the FACT-G total score in the PRISMS patients compared to their UC counterparts, were likely related to study completion. PRISMS provided integral support and education in terms of symptom/complication monitoring and management as well as function recovery during post-ostomy creation recovery, and thus, the PRISMS participants may need time develop self-management confidence and become independent upon study completion. This is significant because we conducted the study during the COVID-19 pandemic when patients and their families had limited access to their treatment team and other supportive care resources. The UC participants only had the printed information to guide post-ostomy self-care from the time of ostomy creation, and thus, had longer time of post-surgical adjustment. We plan to strengthen our wrap-up encounter between the WOCN and PRISMS participants to enhance their confidence and facilitate the transition to self-management. In addition, based on the feedback from our clinical providers, patients, and caregivers, we will expand the follow-up from 60 to 90 days post-surgery in our future efficacy trial so patients and caregivers have the reassurance and support to continue managing the risks of complications and readmission.

The promising results of the preliminary effects indicate that PRISMS has the potential to improve the QOL of patients and caregivers during post-surgical care transition. Although the results of the group differences in QOL, symptoms, and caregiver burden were mostly nonsignificant, there is a positive trend over time among PRISMS patients in the FACT-G subscale scores of physical and emotional well-being among patients and caregivers, respectively. There was also a downward trend in the use of healthcare services and in caregiver burden in the PRISMS patients and caregivers, respectively. Per our analysis of the survey and post-exit interview data, participants reported that the UC education pamphlets were technical, lacked information tailored to patient needs, and did not provide sufficient demonstrations of self-management skills that patients and caregivers could access when needed. Although the research nurse was available to both PRISMS and UC participants, our post-hoc analysis indicated that PRISMS participants contacted the research nurse more frequently than the UC participants, which was likely triggered by the PRISMS education (including text and video demonstrations), skills training, monitoring, and highly tailored and personalized self-management information, all of which was written in plain language and available on-demand. These PRISMS components helped participants recognize symptoms and potential complications and facilitated communication with the research nurse and/or their own treatment team when the symptoms and complications were mild and/or during early onset. Participants reported that PRISMS was useful in the surveys and beneficial during the post-exit interviews. PRISMS participants especially liked the interaction with the WOCN compared to the UC participants whose contact with the WOCN was based on their willingness. Although the group differences in patient healthcare utilization were not statistically significant, one UC patient passed away before completing T2 due to failure to thrive. Thus, PRISMS addressed the care needs of ostomates and their caregivers by providing comprehensive support for symptom- and complication-management. Using an eHealth platform, PRISMS empowers ostomates and caregivers by providing easy and equal access to on-demand, critical education, skills training, monitoring, and self-management information. PRISMS also allows healthcare professionals to triage care for ostomates based on symptom and complication severity and reduce burdens on healthcare professionals. Barriers that challenge PRISMS use include low internet penetration, limited availabilities of digital devices, and low digital and health literacy in low-resource settings. However, compared to current practices, using PRISMS will enable WOCNs to provide the highly specialized care more efficiently and to more patients who may otherwise have to travel long distance to access wound and ostomy care, and thus, expanding their critical role from the hospital to homes in low-resource communities. The increased accessibility, educational content, and enhanced communication may increase patient and caregiver awareness of health and post-surgical changes during the most crucial time of post-ostomy recovery (the first 60–90 days) and reduce the severity of preventable post-ostomy symptoms and complications, which optimizes value-based care delivery. We caution that a confirmatory interpretation of the initial efficacy findings reported in this study should be avoided, due to potential imbalance between UC and PRISMS participants because of the small sample size during the COVID-19 pandemic. However, there is a need for a definitive trial with sufficient power to test the effects of PRISMS on the health outcomes of cancer patients and their family caregivers.

## Limitations

This study has the following limitations. First, it focused on patients with caregivers because caregiver availability is a mandate for discharge. However, some patients were ineligible because they lacked or had minimal caregiver support after discharge. Future research needs to bolster post-operative care strategies for cancer patients who independently manage ostomy and related care. Second, this proof-of-concept pilot RCT had a small sample size, in which most participants were white males, and most caregivers were females. The small sample size also negated the possibility of controlling for the confounding factors in the initial efficacy testing. A future RCT needs sufficient power to examine the effects of PRISMS in cancer patients and caregivers with diverse sociodemographic backgrounds. An adequately powered RCT would also allow for sub-group analyses and identify patients who would benefit the most from the program. Last, we only focused on testing PRISMS for patient-caregiver dyads and excluded patients without a caregiver. Currently, PRISMS is designed in a way that content and functionalities for patients and caregivers could be independent of each other. In other words, for patients without a caregiver or with only minimal support, PRISMS could provide the same education and skills training, personalized feedback based on the continuous monitoring of PRO and digital devices, professional support, as well as social support with other patients. Although it was not the scope of the current study, testing the use of PRISMS for patients without a caregiver is possible in the future.

## Strengths

Despite the small sample size, the current study has the following strengths. First, participants in the PRISMS and UC groups were balanced with regards to demographics and baseline QOL, symptoms, and caregiver burden. Second, PRISMS was a multilevel eHealth intervention that involved patients with cancer, their caregivers, a triage WOCN, and a surgical team. PRISMS provided personalized self-management guidance based on the severity of patient symptoms and complications and utilized wearable devices to facilitate symptom monitoring. Despite the significant impacts of the COVID-19 pandemic, we achieved enrollment and retention rates comparable to existing family-based research. The findings of the feasibility, usability, and acceptability of PRISMS in this rigorous pilot trial laid the foundation for a sufficiently powered RCT.

## Conclusions

This pilot study suggests that our eHealth PRISMS program, which provides tailored and personalized self-management support based on data collected from various sources, is acceptable and usable for patients transitioning from professional care for ostomy creation to in-home self/family-management and their caregivers. A sufficiently powered RCT is needed to test its efficacy in improving the health outcomes of patients and caregivers during the post-ostomy creation care transition.

## Supplementary Information

**Additional file 1.**

## Data Availability

The data that support the findings of this study are available from the University of North Carolina at Chapel Hill, but restrictions apply to the availability of these data, which were used under license for the current study, and so are not publicly available. Data are however available from the authors (lsong@unc.edu) upon reasonable request and with permission of the University of North Carolina at Chapel Hill.

## Abbreviations

QOL: Quality of Life
PRISMS: Patient Reported Outcomes-Informed Symptom Management System
UC: Usual care
RCT: Randomized controlled trial
WOCN: Wound, ostomy, and continence nurse
PRO: Patient-reported outcome
FACT-G: Functional Assessment of Chronic Illness Therapy General Scale
PROMIS: Patient-Reported Outcome Measurement Information System
