# Supplementary material for: An eHealth symptom and complication management program for cancer patients with newly created ostomies and their caregivers (Alliance): a pilot feasibility randomized trial
Source: BMC Cancer. 2023 Jun 10;23:532. doi: 10.1186/s12885-023-10919-x (PMC10257159; doi:10.1186/s12885-023-10919-x)
Supplement: Supplementary file 1 — Additional file 1. [file 12885_2023_10919_MOESM1_ESM.docx]

Appendix 1

Survey of the Usability of PRISMS Program

Please let us know how much you agree with each of the following statements.

Paet1 General (1=Strongly Disagree; 5=Strongly Agree)

1. I thought that PRISMS was easy to use.

1 2 3 4 5

2. The PRISMS has a very attractive presentation.

1 2 3 4 5

3. The PRISMS is both interesting and engaging.

1 2 3 4 5

4. I needed to learn a lot of things before I could get going with PRISMS.

1 2 3 4 5

5. I would imagine that most people would learn to use PRISMS very quickly.

1 2 3 4 5

Paet2 Content (1=Strongly Disagree; 5=Strongly Agree)

1. The content on the PRISMS is written in clear and simple language.

1 2 3 4 5

2. The content is easy to understand and follow.

1 2 3 4 5

3. The content is of high quality.

1 2 3 4 5

4. The content is highly relevant to me.

1 2 3 4 5

5. The online discussion forum is easy to use.

1 2 3 4 5

Paet3 Navigation (1=Strongly Disagree; 5=Strongly Agree)

1. I found what I was looking for quickly and easily.

1 2 3 4 5

2. I found PRISMS unnecessarily complex.

1 2 3 4 5

3. PRISMS did not always do what I expected it to do.

1 2 3 4 5

4. The PRISMS is easy to navigate.

1 2 3 4 5

5. I did not know how to find what I was looking for.

1 2 3 4 5

6. The organization of the PRISMS seems quite logical.

1 2 3 4 5

7. I felt that I had to click too many times to complete typical tasks on the PRISMS

1 2 3 4 5

8. The PRISMS responds quickly.

1 2 3 4 5

9. Using the PRISMS is frustrating.

1 2 3 4 5

For PRISMS user only (1=Strongly Disagree; 5=Strongly Agree)

1. The scheduled monitoring was easy to use

1 2 3 4 5

2. The feedback figures and tips are easy to understand and follow

1 2 3 4 5

3. Contacting nurse(s) is easy

1 2 3 4 5

4. Would you recommend this program to other patients and caregivers?

1 2 3 4 5
